# Comparing the Net Benefits of Adult Deceased Donor Kidney Transplantation for a Patient on the Preemptive Waiting List vs a Patient Receiving Dialysis

**DOI:** 10.1001/jamanetworkopen.2022.23325

**Published:** 2022-07-22

**Authors:** Bryce A. Kiberd, Karthik K. Tennankore, Amanda J. Vinson

**Affiliations:** 1 Division of Nephrology, Department of Medicine, Dalhousie University, Halifax, Nova Scotia, Canada

## Abstract

**Importance:**

Preemptive kidney transplantation is the preferred treatment for end-stage kidney disease. However, deceased donor (DD) kidneys are limited, and the net benefit of allocating kidneys to a preemptively waitlisted patient rather than to a patient receiving dialysis is unclear.

**Objective:**

To estimate the net benefit and costs of allocating kidneys to preemptively waitlisted patients vs those receiving dialysis.

**Design, Setting, and Participants:**

This medical decision analytical model used data from the 2020 US Renal Data System to calculate patient survival among waitlisted patients who received a DD kidney transplant. Four patients were simulated, with similar characteristics: (1) a patient on the preemptive waiting list receiving a DD transplant, (2) a patient on the preemptive waiting list never receiving a transplant, (3) a waitlisted patient already receiving dialysis (dialysis vintage <1 year) receiving a transplant, and (4) a waitlisted patient already receiving dialysis (dialysis vintage <1 year) never receiving a transplant. Annual probability of initiating dialysis (for patients 1 and 2) and duration of dialysis (for patients 3 and 4) were varied in sensitivity analyses.

**Exposures:**

Allocating a DD kidney to a patient on the preemptive waiting list vs the same kidney to a patient receiving dialysis for less than 1 year, with similar recipient characteristics.

**Main Outcomes and Measures:**

Differences in projected quality-adjusted life-years (QALYs) and total costs.

**Results:**

In a simulated patient with a mean start age of 50 years (range, 30-64 years), the patient receiving a preemptive DD transplantation experienced 10.58 (95% CI, 10.36-10.80) QALYs, and the patient on the preemptive waiting list never transplanted experienced 6.83 (95% CI, 6.67-6.99) QALYs. The patient receiving DD transplantation after less than 1 year of dialysis experienced 10.33 (95% CI, 10.21-10.55) QALYs, and the patient receiving dialysis who remained on the waiting list experienced 6.20 (95% CI, 6.04-6.36) QALYs; allocating a DD kidney to the preemptive patient added 3.75 (95% CI, 3.57-3.93) QALYs, whereas allocating the kidney to the patient already receiving dialysis added 4.13 (95% CI, 3.92-4.31) QALYs. While the estimated posttransplant survival was longest for the preemptive transplant recipient, preferentially allocating the kidney to the preemptive patient results in 0.39 (95% CI, 0.49-0.29) fewer QALYs. The net cost of preemptive transplantation was $54 100 (95% CI, $44 100-$64 100) more than transplantation to a waitlisted patient. If the rate of transitioning to dialysis was 20 (rather than 33) events per 100 patient waiting list–years, the net QALYs were −0.67 (95% CI, −0.78 to −0.56). If the patient was receiving dialysis for 3 to 4 years (vs <1 year) the net benefit was not significantly different; however, net costs were considerably higher for the preemptive option.

**Conclusions and Relevance:**

In this decision analytic model study, although allocating DD kidneys to patients preemptively was the best option from a patient perspective, allocating DD kidneys to patients receiving dialysis was a better use of a scare resource from a societal perspective.

## Key Points

**Question:**

How does allocating a deceased donor kidney to a patient on the preemptive waiting list compare with allocating to a patient already receiving dialysis in terms of quality-adjusted life years (QALYs) and cost?

**Findings:**

In this decision analytical model, net QALYs were 0.39 less and net costs were $54 100 more when a deceased donor kidney was allocated to a patient on the preemptive waiting list vs to someone already receiving dialysis.

**Meaning:**

These findings suggest that advocating for greater preemptive deceased donor kidney transplantation at a time when many patients are receiving dialysis and waiting for transplantation may result in fewer net QALYs and greater cost from a societal perspective.

## Teaser

This decision analytical model estimates the net benefit and costs of allocating kidneys to preemptively waitlisted patients vs those already receiving dialysis.

## Introduction

In eligible patients with end-stage kidney disease (ESKD) who desire transplant, preemptive kidney transplantation is considered the optimal form of kidney replacement therapy. Transplanting patients preemptively with a live donor kidney (ideally) or a deceased donor (DD) kidney is associated with better outcomes for patients.^1^ Preemptive transplantation avoids the morbidity and mortality associated with declining kidney function and the institution of dialysis. Furthermore, it avoids the need for access creation. Finally, studies show that patients transplanted preemptively have better graft survival compared with those who are transplanted after the initiation of dialysis,^2^ and it is well known that longer pretransplantation dialysis exposure is associated with inferior outcomes.^3^ In the United States, a patient can be waitlisted preemptively if their estimated glomerular filtration rate (eGFR) is 20 mL/min/1.73 m^2^ or less.^4^

However, demand for a DD kidney transplant is high relative to DD organ supply.^5^ The waiting list is especially long in some donor service areas.^5^ Allocating a kidney to a person on the preemptive list equates to someone who is already receiving dialysis not receiving the transplant. The costs and morbidity of dialysis are high, and removing someone from dialysis provides a significant net benefit.^6,7^ There are also ethical considerations; patients waitlisted preemptively tend to be disproportionately White and well-educated and have private insurance compared with those referred after the start of dialysis.^8,9^ Patients receiving a DD preemptive transplant also tend to receive better-quality organs.^8^ Proposed solutions for disparate access to the preemptive waiting list for marginalized populations include more aggressive and equitable listing through education and innovative electronic medical record capabilities, standardization of waitlisting eligibility criteria (including elimination of race-corrected kidney function estimation), and consideration of eliminating preemptive waitlisting altogether.^9,10^

In addition to these very important ethical concerns, it is unclear whether preemptive DD transplantation is beneficial from a societal perspective. The purpose of this analysis is to estimate the net benefit and costs of allocating kidneys to the preemptive waiting list rather than to those already receiving dialysis. Our hypothesis was that when considering recipient and donor characteristics, transplanting a patient from the preemptive waiting list may provide less net benefit and more costs than transplanting someone who is already receiving dialysis. This study’s findings may inform policy development in the United States.

## Methods

Research ethics approval and the requirement for informed consent were waived by the Nova Scotia Health Authority Research Ethics Board. Reporting followed the Consolidated Health Economic Evaluation Reporting Standards Checklist (CHEERS) reporting guideline.

The purpose of this study was to examine patient outcomes and costs comparing a strategy that diverts DD kidney organs from eligible candidates receiving dialysis toward those on the preemptive waiting list. Patients on the preemptive waiting list who receive a DD transplant are often different from those already initiated on chronic dialysis, including differences across demographic characteristics and comorbid conditions. Therefore, this medical decision analytical model assumes that patients are of similar case-mix for each option.^8,11^

The study examined 4 patients with similar characteristics: (1) a patient on the preemptive waiting list receiving a DD transplant, (2) a patient on the preemptive waiting list never receiving a transplant, (3) a waitlisted patient already receiving dialysis (dialysis vintage <1 year) receiving a transplant, and (4) a waitlisted patient already receiving dialysis (dialysis vintage <1 year) never receiving a transplant. We made a number of key assumptions. First, the DD kidney transplanted is the same for both patients. In a sensitivity analysis, the organ quality (Kidney Disease Risk Index) could be variable, but the same organ would be used. Second, patients in all cohorts are similar in terms of age, sex, race and ethnicity, disease, comorbidity status, and insurance. Third, mortality among patients on the preemptive waiting list is high relative to mortality among patients receiving preemptive DD transplant.^12,13^ Fourth, mortality among patients who received a DD transplant after undergoing dialysis for less than 1 year is equal to the mortality among patients receiving a DD transplant preemptively.^11^ Mortality among patients who received a transplant after undergoing dialysis for more than 1 year is assumed be higher and increased with time spent receiving dialysis.^14^ Fifth, death-censored graft loss in a recipient who has been on dialysis is assumed to be 1.25-fold higher than death-censored graft loss in a preemptive recipient.^11^ Sixth, preemptive patient and graft survival is independent of eGFR at the time of transplantation.^13^ Finally, the remaining years of life were calculated as time under patient survival curves. The net benefit of transplantation was calculated by the difference in area between the patient survival curves.^15^

**Figure 1.**
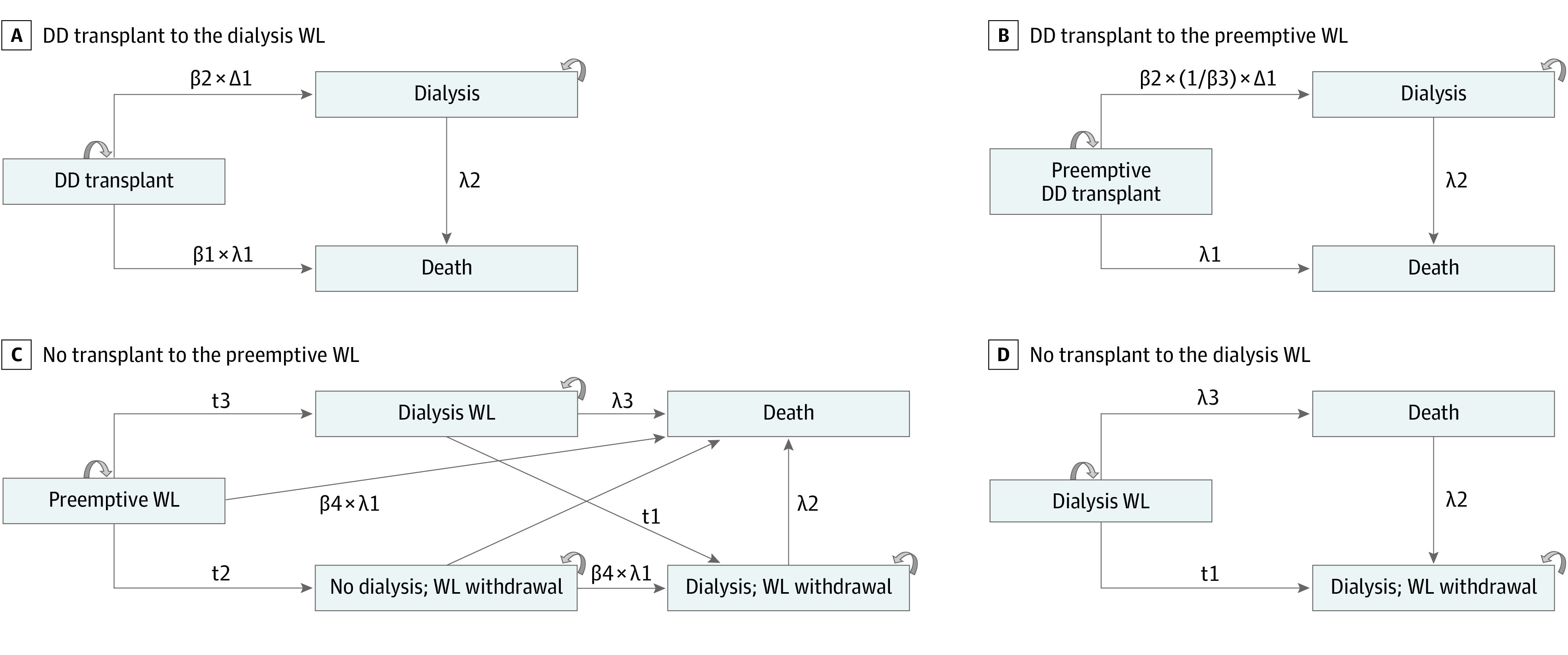
Model Tree for the Study Cohorts Transition variables are λ1, age-adjusted transplant mortality; λ2, age-adjusted waiting list (WL) mortality; λ3, age-adjusted dialysis mortality (failed transplant); t1, age-adjusted withdrawal from the dialysis WL; t2, age-adjusted withdrawal from the preemptive WL; t3, transition rate from preemptive WL to dialysis; and Δ1, age-adjusted graft loss rate. Transition coefficients are β1, increase in transplant mortality if exposed to dialysis; β2, increased risk of graft loss by organ quality; β3, increased risk of graft loss by dialysis exposure; β4, increased mortality while on the preemptive WL (reference, preemptive transplant mortality).

Transition states are shown in Figure 1. To summarize, patients who received a DD transplant (from the dialysis or preemptive waiting list groups) could die with a functioning transplant or die after returning to dialysis. The patient receiving dialysis could die while on the waiting list or be taken off the waiting list and die at some time later. The patient on the preemptive waiting list could die while on the waiting list, be taken off the waiting list and die before starting dialysis, or start dialysis and either remain on the waiting list, be withdrawn, or die.

Annual mortality rates for waitlisted patients receiving dialysis, patients with a functioning transplant, and patients after a failed transplant (return to dialysis) were taken from the US Renal Data System (USRDS) registry database.^16^ Mortality rates for patients on the preemptive waiting list and who had received a DD transplant preemptively were taken from the literature.^12,13^ The time horizon was truncated at age 100 years.^16^ Table 1 shows the additional input variables for the model in addition to the key assumption variables.^11,12,13,16,17,18,19,20,21,22^ Rates of withdrawal from the waiting list were taken from the literature and were age adjusted.^17^ Patients withdrawn from the waiting list were presumed to have a higher mortality rate than those who were active on the waiting list and an equivalent mortality rate to those who returned to dialysis with graft failure.^16^ Given that withdrawal rates are likely to correlate with mortality, we assumed that withdrawal rates from the dialysis waiting list would be higher than those on the preemptive waiting list.

**Table 1.** Input Variables in the Model

Variable	Base case	Range	Distribution^a^	Source
Start age, y	50	30-64	NA	NA
Mortality among those not receiving dialysis				
Transplant, λ1	Age adjusted	NA	NA	USRDS,^16^ 2020
Dialysis exposure, β1^b^	×1.0	0.9-1.1	Lognormal	Grams et al,^11^ 2013
Preemptive WL, with preemptive transplant as reference, β4	×1.6	1.2-2.0	Lognormal	Fissell et al,^12^ 2012; Grams et al,^13^ 2011
Mortality among those receiving dialysis^c^				
Dialysis WL, λ3	Age-adjusted	NA	Gamma	USRDS,^16^ 2020
Dialysis after failed transplant or withdrawal from WL, λ2	Age-adjusted	NA	Gamma	
Graft lost				
Overall, Δ1	Age-adjusted	NA	NA	USRDS,^16^ 2020; Grams et al,^11^ 2013; Grams et al,^13^ 2020
Organ quality, β2	×0.77	0.62-1.61	Lognormal	
Graft loss in patient receiving dialysis vs patient from preemptive WL, β3	×1.25	1.16-1.34	Lognormal	
Withdrawal from WL				
Receiving dialysis, t1	Age-adjusted	NA	Gamma	USRDS,^17^ 2019
Preemptive, t2	×0.5	0.0-1.0	NA	Assumed
Transition				
Preemptive WL to dialysis (t3) per 100 patient years	33	15-75	Lognormal	Assumed
Costs, thousands of 2017 US dollars				
CKD for preemptive WL	23.6	10-35	Gamma	USRDS,^16^ 2020
Access	7.9	5-12	Gamma	
Transplant year 1	133.46	100-150	Gamma	
Transplant year >1	27.36	25-35	Gamma	
Graft loss	122.2	100-140	Gamma	
Dialysis	90.29	80-100	Gamma	
Quality of life				
CKD for preemptive WL	0.80	0.73-0.86	Normal	Wyld et al,^18^ 2012
Dialysis	0.71	0.67-0.76	Normal	
Transplant	0.82	0.74-0.90	Normal	
Discount rate	0.03	0.0-0.05	NA	Neumann et al,^19^ 2016

Abbreviations: CKD, chronic kidney disease; NA, not applicable; USRDS, US Renal Data System; WL, waiting list.

^a^ Density distributions for relative risks were lognormal and were taken from a previous analysis.^20^ Density distributions for costs were gamma.^21^

^b^ Assumes transplant mortality for deceased donor transplantation from the dialysis WL (dialysis duration, <1 year) is equivalent (1.00) to transplant mortality for a preemptively waitlisted patient.^11^ For patients transplanted after 1 to 2, 2 to 3, 3 to 4, and 4 to 5 years of dialysis, the values (for those transplanted from the dialysis waiting list) were 1.10, 1.18, 1.24, 1.35, respectively.^14^

^c^ Mortality on the dialysis WL modeled to increase with time.^16^ Patients transitioning from the preemptive WL to dialysis were modeled to have a higher mortality in the first year.^22^

Graft survival was taken from the USRDS registry.^16^ Annual graft loss rates were calculated from 5-year death-censored graft survival (return to dialysis or repeated transplant). Since graft survival is associated with organ quality, the baseline annual rates of graft loss were adjusted to an overall 5-year graft survival of 81% at 5 years.^13^

The baseline patient was age 50 years (mean age of preemptive patients transplanted with a DD kidney).^11^ The analysis excluded patients transplanted at older than 65 years because several of the assumptions were different and most of the preemptive recipients would be younger than 65 years.^11^

In a series of sensitivity analyses, different rates of transition from the preemptive list to dialysis were examined. To simplify the concept, we assumed that eGFR correlated with likelihood of needing dialysis.^23^ At baseline, we assumed that the rate of requiring dialysis was 33 per 100 patient waiting list–years (33%) for an eGFR of 10 to 14 mL/min/1.73 m^2^, with a range of rates from 20% (eGFR, 14-16 mL/min/1.73 m^2^) to 60% (<10 mL/min/1.73 m^2^).^23^ We also included a rate of 15% to correspond with waitlisted patients with an eGFR of greater than 16 to 20 mL/min/1.73 m^2^.^23^ For the waitlisted patient receiving dialysis, different durations of time receiving dialysis (1-2, 2-3, 3-4, and 4-5 years) were examined.

Quality-adjusted life-years (QALYs) were calculated by applying utility scaling factors for the varying health states.^18^ Costs for the health states were taken from the literature (Table 2). QALYs and costs were 2017 constant US dollars, discounting at 3%.^19^ Costs were from the perspective of the US government (Medicare and Medicaid). Costs for dialysis and transplantation were taken from the USRDS report and did not include noninsured or out-of-pocket patient cost responsibilities.^16^ Costs for chronic kidney disease (CKD) predialysis treatment were also taken from the 2020 USRDS report (stage 4 CKD without congestive heart failure).^16^ Access costs for those on the preemptive waiting list transitioning to dialysis were approximated by taking the total cost of access payments divided by the annual number of procedures (fistula, graft, hemodialysis, and peritoneal catheter placement).^16^ Donor organ acquisition, recipient registry fees, and recipient workup were not included because all groups would have incurred these costs. Net cumulative cost differences were calculated between the 2 options.

**Table 2.** Main Outcomes

Group	Benefit, QALY	Cost, thousands of 2017 US dollars	Net benefit, QALY	Net cost, thousands of 2017 US dollars
**Patients by duration of dialysis**				
<1 y				
No DD transplant	6.20	788.8	4.13 (3.95 to 4.31)	−243.2 (225.2 to 261.1)
DD transplant	10.33	545.6		
3-4 y				
No DD transplant	5.92	753.5	3.74 (3.56 to 3.92)	−235.1 (217.2 to 253.0)
DD transplant	9.66	518.4		
**Patients on preemptive waiting list by probability of initiating dialysis**				
33%/y				
No DD transplant	6.83	726.5	3.75 (3.57 to 3.93)	−189.1 (172.5 to 205.7)
DD transplant	10.58	537.4		
60%/y				
No DD transplant	6.61	771.3	3.97 (3.80 to 4.14)	−197.9 (180.8 to 215)
DD transplant	10.58	537.4		
20%/y				
No DD transplant	7.11	675.6	3.47 (3.29 to 3.65)	−102.2 (86.2 to 118.2)
DD transplant	10.58	537.4		
**Comparison between waiting lists, probability of initiating dialysis vs duration of dialysis**				
33%/y vs <1 y	NA	NA	−0.39 (−0.49 to −0.29)	54.1 (44.1 to 64.1)
60%/y vs <1 y	NA	NA	−0.16 (−0.25 to −0.07)	9.2 (1.0 to 17.4)
20%/y vs <1 y	NA	NA	−0.67 (−0.78 to −0.56)	104.9 (88.7 to 121.1)
33%/y vs 3-4 y	NA	NA	0.01 (−0.13 to 0.15)	45.9 (32.7 to 59.1)
60%/y vs 3-4 y	NA	NA	0.24 (0.10 to 0.38)	1.1 (−7.1 to 9.8)
20%/y vs 3-4 y	NA	NA	−0.27 (−0.40 to −0.14)	96.8 (88.6 to 105.0)

Abbreviations: DD, deceased donor; NA, not applicable; QALY, quality-adjusted life-year.

### Statistical Analysis

The Markov model was developed in TreeAge Pro Healthcare version 2019 R2.1 (TreeAge). Rates were converted to probabilities. The cycle length was yearly. Uncertainty in the net difference between options for costs and outcomes was examined by Monte Carlo microsimulation (2000 trials) to calculate 95% CIs. For statistical comparisons, a 2-tailed *P* < .05 was deemed the threshold for statistical significance.

## Results

In a simulated patient with a mean start age of 50 years (range, 30-64 years), the preemptive DD transplant recipient experienced 10.58 (95% CI, 10.36 to 10.80) QALYs, and the preemptive patient never transplanted experienced 6.83 (95% CI, 6.67 to 6.99) QALYs. This resulted in a gain of 3.75 (95% CI, 3.57 to 3.93) QALYs. The DD transplant patient from dialysis experienced 10.33 (95% CI, 10.21 to 10.55) QALYs, and the dialysis patient that remained on the waiting list experienced 6.20 (95% CI, 6.04 to 6.36) QALYs, for a net gain of 4.13 (95% CI, 3.92 to 4.31) QALYs. Preferential preemptive transplantation resulted in −0.39 (95% CI, −0.59 to −0.29) QALYs and, as shown in Table 2, resulted in a net added cost of $54 100 (95% CI, $44 100 to $64 100) compared with allocation to a waitlisted patient already receiving dialysis.

If the preemptive patient was less likely to need dialysis (20% per year), the difference was −0.67 (95% CI, −0.78 to −0.56) QALYs at a higher net added cost of $105 900 (95% CI, $89 700 to $122 100). If the patient was more likely to need dialysis (60% per year), the net benefit was −0.16 (95% CI, −0.25 to −0.07) QALYs, at a higher net cost of $9200 (95% CI, $1000 to $17 400).

If the patient in the dialysis group had been receiving dialysis for 3 to 4 years, they experienced 9.66 (95% CI, 9.44 to 9.88) QALYs, and the patient in the dialysis group who remained on the waiting list experienced 5.92 (95% CI, 5.76 to 6.08) QALYs. In this case, the net benefit for the patient receiving preemptive transplant was 0.01 (95% CI, −0.13 to 0.15) QALYs, with a net cost of $45 900 (95% CI, $32 700 to $59 100) compared with allocation to the dialysis waiting list. If the preemptive patient was more likely to need dialysis (60% per year), the net benefit favored preemptive transplantation (0.24 [95% CI, 0.10 to 0.38] QALYs). However, if the preemptive patient was less likely to need dialysis (20% per year), there was a loss of net benefit with preemptive transplantation (−0.27 [95% CI, −0.40 to −0.14] QALYs).

**Figure 2.**
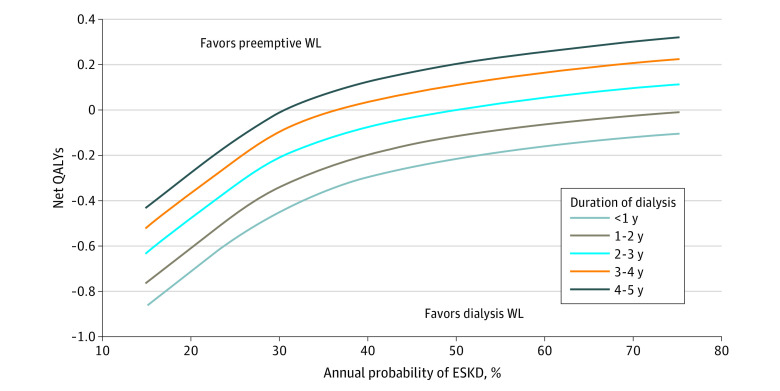
Net Benefit of Dialysis Vintage vs Probability of End-Stage Kidney Disease (ESKD) QALY indicates quality-adjusted life-years; WL, waiting list.

**Figure 3.**
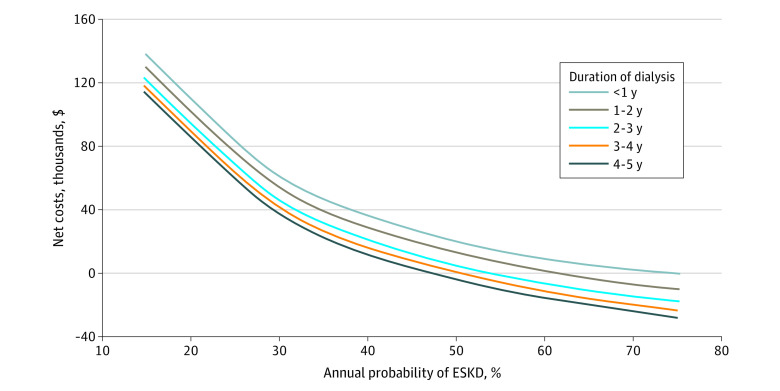
Net Costs of Dialysis Vintage vs Probability of End-Stage Kidney Disease (ESKD)

Figures 2 and 3 show the net benefit and net costs, respectively, in a 2-way sensitivity analysis. If the probability of needing dialysis in a patient in the preemptive group was less than 30% per year, the net benefit favored transplanting the patient already receiving dialysis irrespective of time on dialysis. For preemptively waitlisted patients with a very low annual probability of needing dialysis, the net loss of QALYs was substantial compared with transplanting someone already receiving dialysis. In all scenarios, the net costs were higher for transplanting a preemptively waitlisted patient if the annual probability of needing dialysis was less than 45%.

eFigures 1 and 2 in the Supplement show Tornado diagrams for the variables. For net benefit, allocating to the dialysis waiting list (with dialysis duration <1 year) was more favored if the quality of life in the preemptive health state was higher than our baseline assumptions and mortality on the preemptive waiting list was lower than our baseline assumptions. Lower annual costs for preemptively waitlisted patients and higher annual dialysis costs would add to the costs of allocating a DD kidney to the patient on the preemptive waiting list.

## Discussion

In this study, we found that allocating a DD kidney organ to a patient on the preemptive waiting list rather than someone already receiving dialysis may result in fewer net QALYs and greater net costs from a societal perspective. Preemptively waitlisted patients who are transplanted had longer estimated patient survival compared with those who are transplanted from the dialysis waiting list. Patients with longer dialysis exposure had even shorter posttransplantation survival. With an unlimited supply of DD organs, all patients should be transplanted preemptively before they require dialysis or become too ill and need to be removed from the waiting list.

However, this analysis also shows that allocating DD organs to the preemptive waiting list and away from those already receiving dialysis may not be the best use of resources. The study found that the net benefit and costs of kidney transplantation favored allocating the DD organ to the patient on the dialysis waiting list if they had been receiving dialysis for less than 1 year. DD preemptive transplantation was favored if allocation was to a person on dialysis for 4 or more years; this was not the case if the preemptively waitlisted patient had a low likelihood of needing dialysis. Community and health care preferences weigh transplantation toward those with more severe illness, those who have been waiting longer for a transplanted organ, and those who would experience greater net benefit.^24^ Patients receiving dialysis have lower quality of life than those not yet receiving dialysis.^18^ Patients who have been receiving dialysis for longer periods of time have even higher mortality rates.^16^

The study found that the likelihood a patient on the preemptive waiting list would need dialysis is an important factor when determining net benefit. From a societal perspective, the ideal time to transplant a patient is just before they need dialysis. This study supports the recent Kidney Disease Improving Global Outcome clinical practice guideline to limit preemptive DD transplantation in adult patients to when the eGFR is less than 10 mL/min/1.73 m^2^. As recently reviewed, some countries do not permit access to the transplant waiting list until the patient is already receiving dialysis.^9^ Some allow preemptive listing only if dialysis is imminent.^9^ Unfortunately, timing transplantation at the brink of needing dialysis is not likely to be feasible for a DD organ, and completely eliminating preemptive wait listing may result in delayed access to transplantation for patients who eventually initiate dialysis.

Allocating a DD organ to a preemptively waitlisted patient with a higher eGFR is likely to generate even fewer net QALYs at greater costs. One of the potential consequences of listing everyone eligible for a transplant at an eGFR of 15 to 20 mL/min/1.73 m^2^ rather than at less than 10 mL/min/1.73 m^2^ is that there could be exponential increases in the preemptive waiting list. In a recent study of patients followed up by the Kaiser Permanente Northern California health care organization, there were 1487 patients without ESKD with an eGFR of less than 10 mL/min/1.73 m^2^, 3274 with an eGFR of 10 to 13 mL/min/1.73 m^2^, and 10 325 with an eGFR of 14 to 19 mL/min/1.73m^2^ in the community of 4.5 million.^23^ The more patients on the preemptive list, the greater the likelihood of diverting organs from the dialysis waiting list cohort. Those undergoing dialysis will wait longer and will be more likely to die or be removed from the list. Acknowledging the complexities of the kidney allocation system, ideally one of the goals of allocation should be to minimize time spent receiving dialysis.

Timing of transplantation from a patient’s perspective should be preemptive as long as death on the preemptive waiting list is higher than death with a functioning transplant. At this time, this threshold is not known and could differ significantly with patient profile and listing eGFR. Studies in patients with liver disease show that transplanting patients with low disease scores is associated with significantly higher risk of death.^25^ The trade-off of a higher eGFR with transplantation and the risks associated with immunosuppression are not clearly defined in preemptively waitlisted patients with an eGFR of 20 mL/min/1.73 m^2^ compared with an eGFR of 10 mL/min/1.73 m^2^.

This analysis is difficult given that patients on the preemptive waiting list are very different from those receiving dialysis and may receive a different quality kidney.^8,11^ For example, a patient who is aged 50 years and has diabetes with an eGFR of 20 mL/min/1.73 m^2^ and a 50-year-old patient with diabetes who has been undergoing dialysis for 3 years are not the same. The latter has had to survive (immortal time bias) multiple years to end up receiving dialysis. These issues hamper direct comparisons between those transplanted preemptively and those transplanted from the dialysis waiting list.^26,27^ Nonetheless, our analysis is consistent with studies that question the benefit and fairness of DD preemptive transplantation.^11,27,28,29^

### Limitations

There are limitations to this analysis. The analysis did not consider individual characteristics, such as race, sex, ESKD cause, insurance, and human leukocyte antigen matching, but rather examined average practice based on age-stratified survival USRDS tables. As such, the findings of this study should be confirmed with patient-level data analysis. The model was constructed to compare similar patients with the same allocated kidney in a decision analysis framework. The study was not intended to determine the ideal threshold for waitlisting patients, given that progression to ESKD would differ based on other factors in addition to eGFR.^30^ The study did not consider the fact that a preemptive patient could be allocated a kidney based on matching criteria that would confer additional benefit. However, that benefit would likely be relatively small and most important to a younger recipient who is less likely to die with a functioning transplant. This study did not suggest preferentially allocating organs to patients receiving dialysis for less than 1 year rather than to those receiving dialysis for more than 1 year.

The perspective of the cost analysis is from the US federal government. In reality, many preemptive DD recipients have private coverage.^9,11,13^ One could argue that transplanting patients with private coverage would save money for the government; however, private insurance payments are often higher than Medicare and Medicaid reimbursements, and eliminating private payments from the model calculations would suggest that costs of transplantation are free.^31,32,33^ Out-of-pocket costs were not included nor was reimbursement for time spent receiving treatment.^34^ The major factors in the cost analysis were the annual costs of dialysis and pretransplant CKD therapy. As mentioned previously, an analysis of costs at an individual patient level would be helpful. It is important to note, however, that the primary limitation of kidney allocation policies is the scarcity of donor organs, not the fiscal costs.

## Conclusions

The implications of this study will be controversial. Preemptive transplantation is well established. However, the findings of this study should challenge the principles behind the practice of waitlisting patients with a low probability of needing dialysis within a short period.^10,35,36^ Preemptive waitlisting may be the very strategy that systematically favors some groups above others.^9^ Rather than eliminating preemptive transplantation, restricting the list to those likely to be on dialysis within a year or who are highly sensitized (>98%) may be an optimal approach, provided equal access can be assured for all. The best solution is greatly increasing the supply of kidney organs. However, this is not likely achievable in the near future. Encouraging preemptive waitlisting for patients with a low likelihood of needing dialysis could be counterproductive, result in exponentially more people waitlisted, incur greater costs, with possibly lower overall societal benefit.

## Notes

Supplement. **eFigure 1. **Tornado Plot for 1-Way Sensitivity Analysis: QALYs Lost per Allocated DD Kidney to a Patient on Preemptive Waiting List vs Patient Receiving Dialysis for Less Than 1 Year **eFigure 2. **Tornado Plot of Cumulative Present Value Costs per Allocated DD Kidney to a Patient on Preemptive Waiting List vs Patient Receiving Dialysis for Less Than 1 Year
