# Supplementary material for: Comparing the Net Benefits of Adult Deceased Donor Kidney Transplantation for a Patient on the Preemptive Waiting List vs a Patient Receiving Dialysis
Source: JAMA Netw Open. 2022 Jul 22;5(7):e2223325. doi: 10.1001/jamanetworkopen.2022.23325 (PMC9308061; doi:10.1001/jamanetworkopen.2022.23325)
Supplement: Supplement. — eFigure 1. Tornado Plot for 1-Way Sensitivity Analysis: QALYs Lost per Allocated DD Kidney to a Patient on Preemptive Waiting List vs Patient Receiving Dialysis for Less Than 1 Year eFigure 2. Tornado Plot of Cumulative Present Value Costs per Allocated DD Kidney to a Patient on Preemptive Waiting List vs Patient Receiving Dialysis for Less Than 1 Year [file jamanetwopen-e2223325-s001.pdf]

Supplemental Online Content

Kiberd BA, Tennankore KK, Vinson AJ. Comparing the net benefits of adult deceased donor kidney transplantation for a patient on the preemptive waiting list vs a patient receiving dialysis. *JAMA Netw Open.* 2022;5(7):e2223325. doi:10.1001/jamanetworkopen.2022.23325

eFigure 1. Tornado Plot for 1-Way Sensitivity Analysis: QALYs Lost per Allocated DD Kidney to a Patient on Preemptive Waiting List vs Patient Receiving Dialysis for Less Than 1 Year

eFigure 2. Tornado Plot of Cumulative Present Value Costs per Allocated DD Kidney to a Patient on Preemptive Waiting List vs Patient Receiving Dialysis for Less Than 1 Year

This supplemental material has been provided by the authors to give readers additional information about their work.

© 2022 Kiberd BA et al. *JAMA Network Open.*

eFigure 1. Tornado Plot for 1-Way Sensitivity Analysis: QALYs Lost per Allocated DD Kidney to a Patient on Preemptive Waiting List vs Patient Receiving Dialysis for Less Than 1 Year

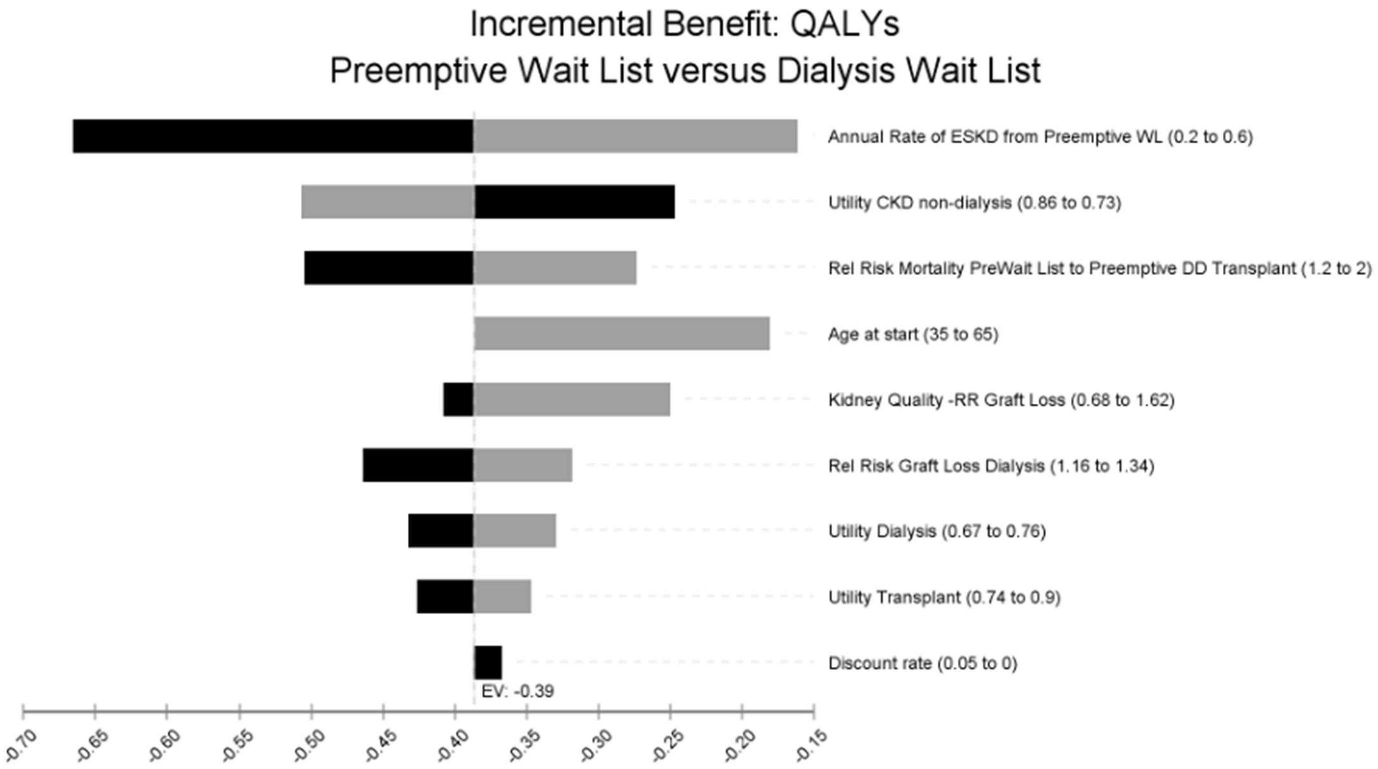

© 2022 Kiberd BA et al. *JAMA Network Open.*

eFigure 2. Tornado Plot of Cumulative Present Value Costs per Allocated DD Kidney to a Patient on Preemptive Waiting List vs Patient Receiving Dialysis for Less Than 1 Year

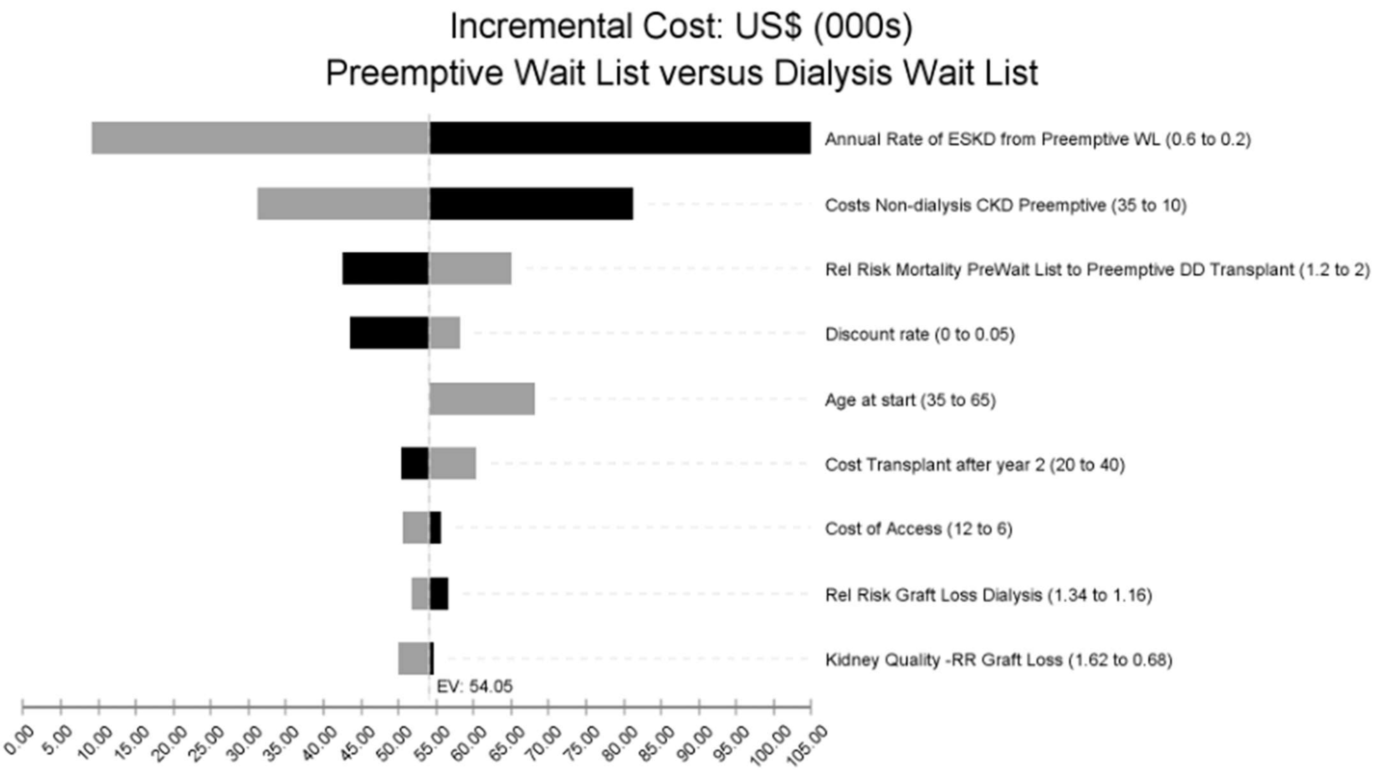

© 2022 Kiberd BA et al. *JAMA Network Open.*
